# Melanocyte Colonization and Pigmentation of Breast Carcinoma: Pathological and Clinical Aspects

**DOI:** 10.1155/2012/427628

**Published:** 2012-12-20

**Authors:** Marco Mele, Tinne Laurberg, Tine Engberg Damsgaard, Jonas Funder, Vibeke Jensen

**Affiliations:** ^1^Department of Endocrine and Breast Surgery, Aarhus University Hospital, Tage Hansens Gade 2, 8000 Aarhus C, Denmark; ^2^Department of Pathology, Aarhus University Hospital, Tage Hansens Gade 2, 8000 Aarhus C, Denmark; ^3^Department of Plastic Surgery, Aarhus University Hospital, Nørrebrogade 44, 8000 Aarhus C, Denmark

## Abstract

*Introduction*. Melanocyte colonization of breast carcinoma by nonneoplastic melanocytes of epidermal origin is a rare and serious condition first described in 1977. We report on the exceptional clinical and pathological features of this migration phenomenon in a 74-year-old patient. *Discussion*. The pathogenesis by which melanocyte migration takes place is not known, but a breached basement membrane is considered essential. *Conclusion*. Histological examination and additional staining of skin are essential to differentiate breast cancer melanosis from malignant melanoma.

## 1. Introduction

Melanocyte colonization of breast carcinoma by nonneoplastic melanocytes of epidermal origin was first described by Azzopardi and Eusebi in 1977 [1]. Tumor cells disrupting the epidermal-dermal interface are considered to be essential for the migration of melanocytes into the superficial dermis. This condition is rare, and the pigmentation is usually only observed microscopically. Pigmented skin lesions in breast cancer patients have been described in recent years. Multiple pigmented lesions developed in a mastectomy scar of a breast cancer patient are reported by Marco et al. [2], and Gadkari et al. describe how the cytological findings of melanocytes colonization in subcutaneous secondary breast carcinoma differ from that of malignant melanoma [3]. A case of cutaneous metastatic breast carcinoma with melanocyte colonization, which mimicks malignant melanoma as described in detail by Wyatt et al. [4]. Furthermore, examples of pigmented mammary Paget disease mimicking malignant melanoma are found in recent literature [5–8].

We report on this exceptional clinical and pathological migration phenomenon and demonstrate that histological examination and additional staining are essential to differentiate malignant melanoma from breast cancer melanosis.

## 2. Materials and Methods

### 2.1. Case

A seventy-four-year-old woman, in good condition, was in August 2009 diagnosed with a large tumor of the breast. It was localized in the left upper quadrant with infiltration of the skin and pectoral muscle as well as massive involvement of the axillary lymph nodes. Computerized tomography showed suspicious distant metastases in the mediastinum and lung.

**Figure 1:**
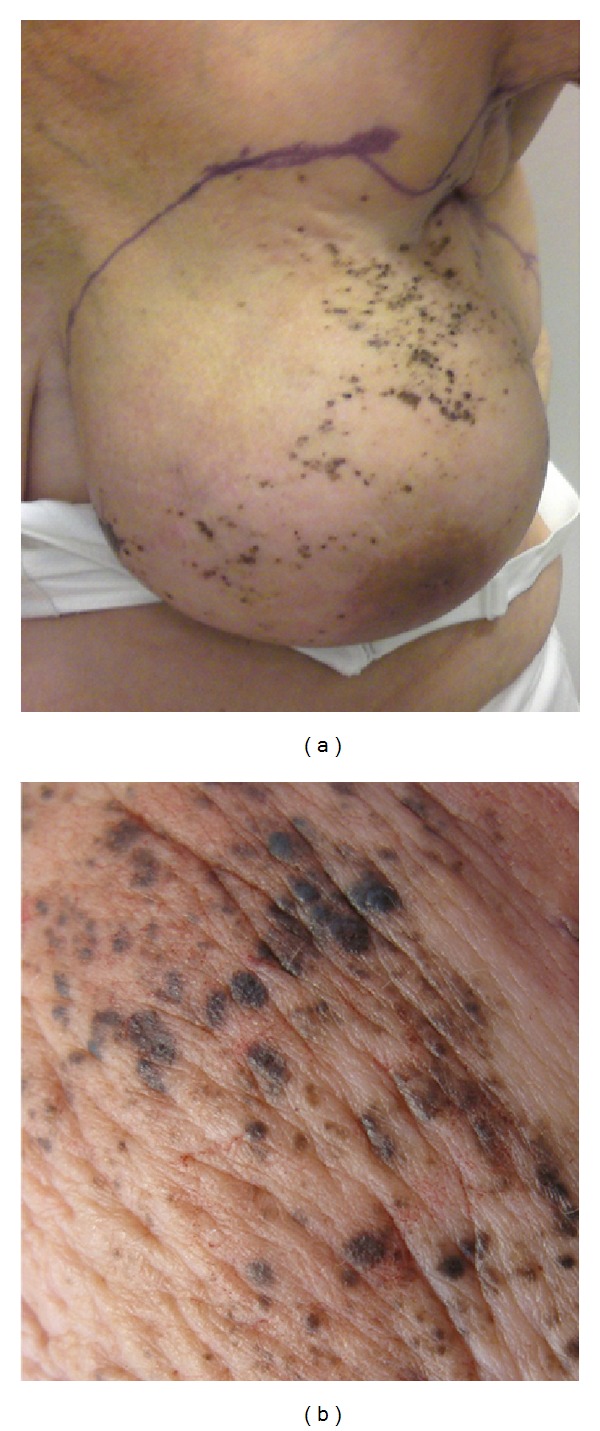
(a) Large numbers of pigmented areas on the skin of the affected breast. (b) Pigmented maculae.

The histological examination after core needle biopsy showed an estrogen receptor positive carcinoma. The patient was initially treated with Letrozole. This treatment induced partial regression of the tumor and metastases. In April 2010 she developed large numbers of heavily pigmented areas on the skin of the affected breast mimicking malignant melanoma, and she was referred to the Department of Plastic Surgery (Figure 1(a)). 

A representative excisional biopsy from the pigmented skin was performed, and histological examination showed carcinoma with no evidence of collision tumor.

As her tumor and metastases had exhibited significant regression upon treatment with Letrozole, she was evaluated at a multidisciplinary conference. It was decided to offer the patient mastectomy with axillary dissection and a V-Y plasty using the latissimus dorsi musculocutaneous flap. 

The patient was discharged after 8 days of hospitalization in good condition and was referred to The Department of Oncology.

### 2.2. Macroscopy

Examination of the mastectomy specimen showed peau d'orange and multiple 1–4 mm heavily pigmented nonelevated areas in the skin (maculae) (Figure 1(b)). 

The mammary tissue contained a more than 10 cm large area of indurated tissue, suggestive of carcinoma with signs of lymphangitis carcinomatosis.

### 2.3. Microscopy

**Figure 2:**
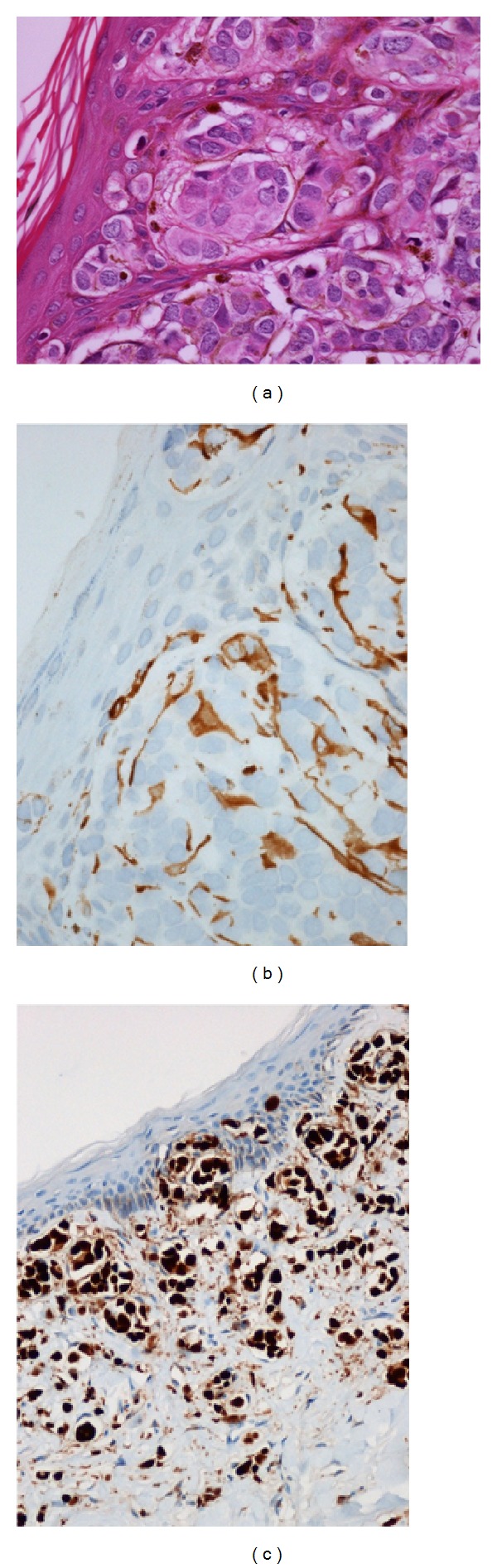
(a) Melanocytes colonizing the underlying carcinoma cells. (HE × 40). (b) Carcinoma cells surrounded by melan-A-positive melanocytes (×40). (c) Estrogen receptor positive carcinoma cells (×20).

Massive vascular tumor embolisation was seen in the entire mammary tissue including the dermis. In several locations, the adenocarcinoma cells had infiltrated the superficial dermal connective tissue and penetrated the dermoepidermal junction (Figure 2(a)). At these specific areas, abundant melanin pigment was observed in elongated and dendritic melanocytes colonizing the underlying carcinoma cells.

Additional immunohistochemical stainings identified positive estrogen receptor (Figure 2(c)) and CK7/19-positive carcinoma cells intermingled with and surrounded by positive melan A (Figure 2(b)) and MIFT-positive cells.

## 3. Discussion and Conclusion

The presence of melanin pigmentation is described in various epithelial tumors like carcinomas of the breast, oral mucosa, larynx, salivary gland, prostate gland, and the rectal mucosa [1, 2, 9–13]. The presence of melanocytes in the mucosa is thought to be the pigment source for melanosis in many of these organs. It is assumed by some authors that transforming growth factors and epidermal growth factor receptors are involved in the proliferation of normal and neoplastic human cells. *In vitro* it has been shown that activated melanocyte growth factor stimulates the proliferation of the normal human melanocytes, and this is thought to be induced by a transforming growth factor present in the breast cancer cells [14]. However, the exact pathogenesis by which melanocyte migration takes place is not fully understood, but a breached basement membrane is probably essential [1].

In the rare cases of visible pigmentation of the skin covering a breast cancer, malignant melanoma should be included in the differential diagnosis. On clinical examination, malignant melanoma most often shows an elevated lesion, whereas the pigmentation of melanosis is at the level of the skin. However, histological examination and additional immunohistochemical staining are essential to differentiate malignant melanoma and breast cancer melanosis.
